# University of Buffalo

**Published:** 1857-08

**Authors:** 


*University of Buffalo — Medical Department.* — With this number appears the annual advertisement of the Buffalo Medical College. A glance at its list of teachers will show no change in its organization, except in the post of Demonstrator of Anatomy. The present arrangement of the curriculum of study, and the division of labor among the different members of the faculty, has proved profitable to the student and pleasant for the teacher. So long as our present imperfect national system of education is continued, we regard this organization of the various chairs as that best suited to the wants of the student.

Of the men who make up this faculty, we could say much; but they are our colleagues and our personal friends, and we must withhold the praise we would otherwise gladly utter. Fortunately, they are men whose names speak for themselves.

Of the other advantages of the school we speak without fear of being misunderstood. We state that which we know, when we say that it affords the best clinical advantages of any school in the United States. Its clinics are located so near to the college edifice, that no student can urge the excuse of inconvenience for absenting himself from them, while, for the large variety of disease presented, we have only to appeal to the surgical and medical clinical reports published from month to month in our pages. Moreover, having good advantages, it uses and profits by them.

The past term witnessed a satisfactory increase in the number of students. Present circumstances indicate a still further improvement in this respect; and those interested in the school feel strong in the belief that, with all the strong competition under which it must labor, it will sustain the hopes of its self-sacrificing founders. To this end are pledged the earnest efforts of the faculty.

